# Development and validation of a robust logistic regression model for predicting osteoporosis in older people

**DOI:** 10.3389/fmed.2026.1840631

**Published:** 2026-07-28

**Authors:** Hongxia Xue, Xuewei Hao, Dandan Liu, Yongqiang Li, Haiquan Yu

**Affiliations:** 1 Health Management Center, The Third Hospital of Shijiazhuang City, Shijiazhuang, Hebei, China; 2 Department of Orthopedics, The People’s Hospital of Shijiazhuang City, Shijiazhuang, Hebei, China; 3 Department of Ophthalmology, The People’s Hospital of Shijiazhuang City, Shijiazhuang, Hebei, China; 4 Department of Health Management Center, The First Hospital of Hebei Medical University, Shijiazhuang, Hebei, China; 5 Department of Orthopedics, The First Hospital of Hebei Medical University, Shijiazhuang, Hebei, China

**Keywords:** machine learning, osteoporosis, predictive model, Random Forest, risk assessment

## Abstract

**Background:**

Osteoporosis (OP) represents a significant public health challenge in the aging population, often leading to debilitating fractures. Early identification of high-risk individuals through routine clinical data is vital for preventive care. This study aimed to develop and validate a robust machine learning-based framework to predict OP in older people using comprehensive physical examination indicators.

**Methods:**

We analyzed a retrospective single-center cohort of 852 participants (age ≧ 60 years) from a physical examination center. The cohort was randomly partitioned into a training set (*n* = 598) and an internal validation set (*n* = 254) at a 7:3 ratio. Nineteen potential predictors, including demographics (Age, Sex, BMI), lifestyle habits, comorbidities, and biochemical markers (e.g., SUA, Hb, LDL), were evaluated. After feature selection via LASSO regression, five algorithms-Logistic Regression (LR), Random Forest (RF), Decision Tree (DT), Support Vector Machine (SVM), and XGBoost-were constructed. Hyperparameter tuning was performed only within the training set using 5-fold cross-validation and grid search, and the internal validation set was kept separate for final performance assessment. Model performance was evaluated using the Area Under the Curve (AUC), calibration curves, and Decision Curve Analysis (DCA). Restricted cubic spline (RCS) analysis was further employed to evaluate dose–response associations between selected variables and osteoporosis risk.

**Results:**

The prevalence of OP in the study population was 28.87% (246/852). LASSO regression identified 12 optimal features for model development. While ensemble methods (RF and XGBoost) exhibited high discriminative power, they showed signs of potential overfitting with perfect training set performance. In contrast, the LR model demonstrated superior robustness and stability, achieving an AUC of 0.809 in the training set and 0.782 in the internal validation set. In the validation cohort, the LR model maintained balanced performance with a sensitivity of 0.767 and a specificity of 0.702. Calibration curves indicated agreement between predicted and observed risks, and DCA suggested clinical net benefit across a broad range of threshold probabilities. SHAP (SHapley Additive exPlanations) analysis indicated that Sex, Age, and BMI were the most influential predictors. Lower levels of serum creatinine (Scr), serum uric acid (SUA), and fasting plasma glucose (FPG) were also associated with higher predicted osteoporosis risk. Multivariable-adjusted RCS analysis suggested an upward trend between HDL-C and OP risk, particularly in the female subgroup (OR = 3.35, 95% CI: 0.86–13.04; *p* = 0.081), but this finding did not reach conventional statistical significance and should be interpreted cautiously.

**Conclusion:**

Compared with complex ensemble algorithms, the LR model provides a stable and interpretable approach for identifying older people who may warrant DXA assessment or closer bone-health evaluation. Because this study used a retrospective single-center cohort and internal validation only, external multi-center validation is required before routine clinical deployment.

## 1 Introduction

Osteoporosis (OP) is characterized by low bone mass, increased bone fragility, and increased susceptibility to fracture, which is associated with substantial morbidity, mortality, and economic costs (1). Many studies indicated that OP has a high prevalence worldwide, particularly among older people (2–4). Despite the availability of Dual-energy X-ray Absorptiometry (DXA) as the diagnostic gold standard, its limited accessibility in primary care and routine physical examination settings often leads to a failure in identifying high-risk individuals before the occurrence of the first fracture (5, 6). The intended role of the present model is therefore not to replace DXA, but to function as a low-cost pre-screening and risk-stratification tool based on routinely collected clinical indicators. In settings where DXA availability is limited, such a model may help clinicians prioritize high-risk older people for DXA referral, lifestyle counseling, fall-prevention education, and targeted follow-up. Therefore, developing a non-invasive, accessible, and high-accuracy screening tool based on routine clinical indicators is important for early intervention in geriatric bone health.

In the past decade, epidemiological research has systematically established profound links between metabolic syndrome components and bone homeostasis, shifting the paradigm of OP from a localized skeletal disorder to a manifestation of systemic metabolic dysregulation (7, 8). Specifically, type 2 diabetes mellitus (T2DM) and its associated chronic hyperglycemia are linked to a unique “bone density-fracture risk” paradox, where fracture risk increases despite relatively preserved bone mineral density (9, 10). Concurrently, dyslipidemia and non-alcoholic fatty liver disease (NAFLD), as indicators of systemic metabolic turbulence, have been identified as independent risk factors for accelerated bone loss (11, 12). Methodologically, machine learning (ML) algorithms—such as Random Forest (RF) and XGBoost—have become the prevailing tools for clinical predictive modeling due to their superior ability to capture complex, non-linear interactions within high-dimensional datasets compared to traditional linear statistics (13). However, a major limitation of complex algorithms is their “black-box” nature, which hinders clinical transparency. Therefore, incorporating model interpretability tools like SHapley Additive exPlanations (SHAP) is essential to bridge the gap between artificial intelligence-driven predictions and clinical decision-making in geriatric bone health (14).

Despite these advancements, several critical gaps persist in existing literature. Most predictive studies focus on fragmented dimensions, such as isolated metabolic markers or specific lifestyle factors, rather than adopting a holistic “metabolic-lifestyle-comorbidity” framework. Furthermore, while systemic low-grade inflammation—often termed “inflamm-aging”—and metabolic dysregulation have been implicated in skeletal deterioration, the integrated predictive utility of candidate markers, such as the Neutrophil-to-Lymphocyte Ratio (NLR), remains controversial and lacks large-scale validation in asymptomatic geriatric cohorts (15). To date, few studies have integrated these multi-dimensional features through a rigorous machine-learning comparison to identify a stable, clinically applicable model specifically tailored for older people.

To clarify the research gap, this study differs from previous machine-learning studies in Chinese populations in three aspects: (1) it focuses specifically on older people undergoing routine physical examinations rather than hospital-based disease cohorts; (2) it integrates demographic, lifestyle, comorbidity, biochemical, and inflammatory indicators into a single screening framework; and (3) it prioritizes a stable and interpretable model suitable for pre-DXA risk stratification instead of selecting the algorithm with the highest apparent AUC alone. Thus, the purpose was not to repeat prior prediction studies, but to evaluate whether routinely available examination data can support a transparent osteoporosis pre-screening tool for an health-examination population of older people.

To address these limitations, the present study developed and validated an optimized ML framework to predict OP risk in a cohort of 852 participants aged ≧60 years. By evaluating a comprehensive panel of 19 clinical variables—encompassing physiological metrics, metabolic markers (e.g., lipid profiles, serum uric acid), and inflammatory indices (e.g., NLR)—we utilized Least Absolute Shrinkage and Selection Operator (LASSO) regression for robust feature selection. We compared five distinct algorithms—Logistic Regression (LR), Random Forest (RF), Decision Tree (DT), Support Vector Machine (SVM), and XGBoost—with a particular emphasis on balancing discriminative power and clinical generalizability. Finally, we employed SHAP analysis to ensure model transparency, providing an interpretable screening solution to facilitate early risk stratification and bone health management in the aging population.

## 2 Methods

### 2.1 Study design and data sources

This study was designed as a retrospective single-center cohort study to develop and internally validate a predictive framework for OP in older people. De-identified clinical data were extracted from the electronic physical examination database of the First Hospital of Hebei Medical University, covering the period from January 2024 to December 2024. The study protocol was conducted in accordance with the Declaration of Helsinki and was approved by the Institutional Ethics Committee of the First Hospital of Hebei Medical University.

The First Hospital of Hebei Medical University is a tertiary hospital located in Shijiazhuang, the capital city of Hebei Province in northern China. The physical examination center mainly serves urban and surrounding suburban residents who attend routine self-paid or employer-organized health examinations. The study population was predominantly Han Chinese, reflecting the demographic composition of the local catchment area; therefore, extrapolation to rural populations, minority ethnic groups, or other regions should be made cautiously.

### 2.2 Study participants and inclusion/exclusion criteria

Initially, a total of 2,750 individuals who underwent routine physical examinations were screened. The inclusion criteria were as follows: (1) aged 60 years or older; (2) availability of complete clinical and biochemical indicators; and (3) confirmed diagnosis of bone status via DXA.

**Figure 1:**
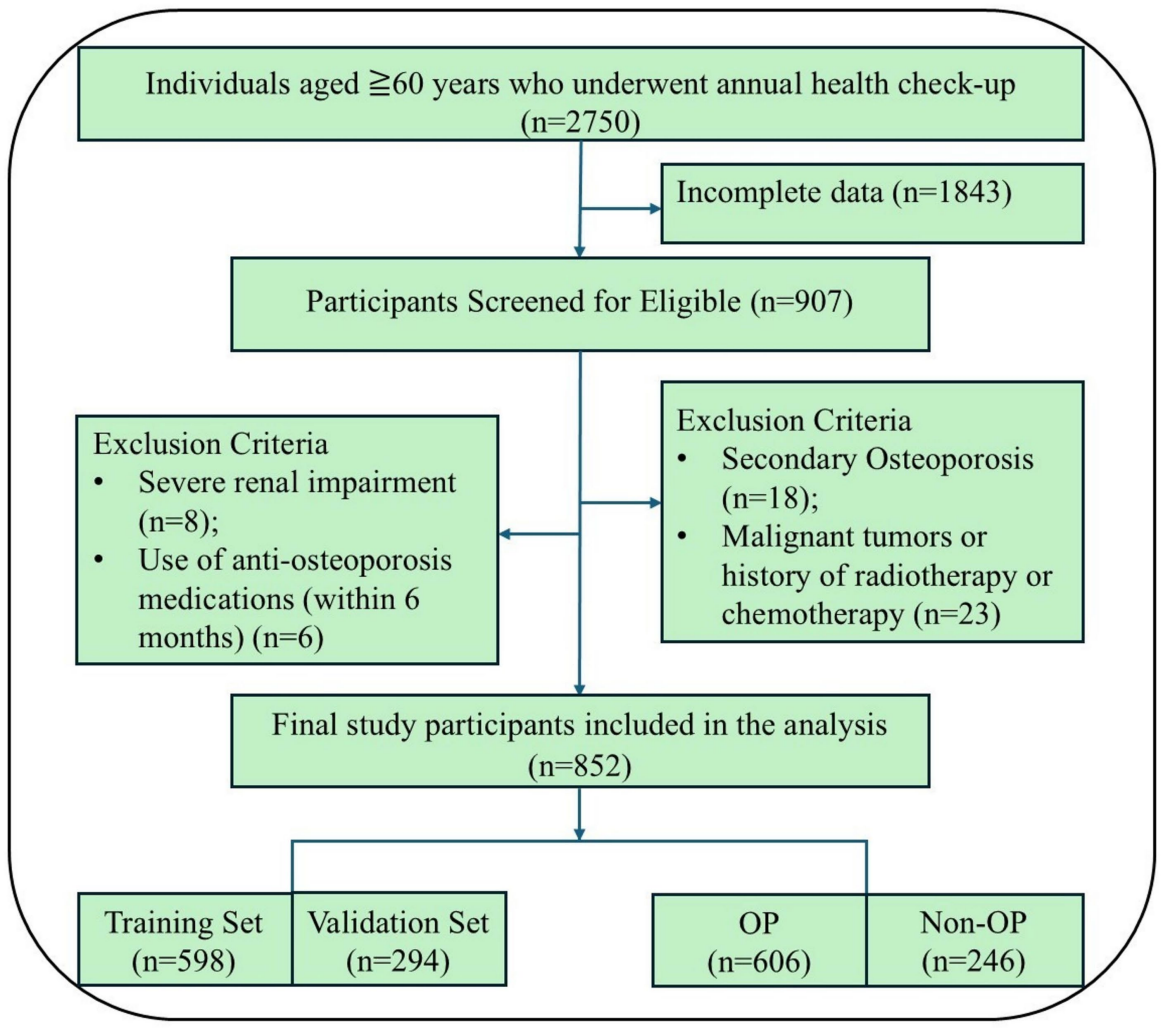
Flowchart of participants recruitment.

Exclusion criteria included: (1) secondary osteoporosis caused by hyperparathyroidism, hyperthyroidism, or long-term use of glucocorticoids; (2) history of malignant tumors; (3) presence of acute infectious diseases during the examination; and (4) records with significant missing data (>5%). Following a rigorous screening process, 852 participants were ultimately enrolled, consisting of 246 patients in the OP group (T-score ≤ − 2.5) and 606 individuals in the non-OP control group. Finally, 852 participants were included in the study (Figure 1).

No formal *a priori* sample size calculation was performed because this was a retrospective analysis based on all eligible participants with complete DXA and clinical data during the study period. Nevertheless, the final dataset included 246 osteoporosis events among 852 participants. With 19 candidate predictors before LASSO selection and 12 predictors retained in the final modelling process, the event-per-variable ratio was approximately 12.9 before selection and 20.5 after selection, which meets commonly used pragmatic requirements for multivariable prediction modelling. We acknowledge that this sample remains smaller than some recent multicenter Chinese studies, and this is now emphasized as a limitation.

### 2.3 Variable definition and data collection

Comprehensive demographic and clinical data were collected for all participants. Physiological Indicators: Age, sex, height, weight, and Body Mass Index (BMI, calculated as weight in kilograms divided by the square of height in meters). Biochemical Markers: Fasting plasma glucose (FPG), total cholesterol (TC), triglycerides (TG), high-density lipoprotein cholesterol (HDL-C), low-density lipoprotein cholesterol (LDL-C), hemoglobin (Hb), alanine aminotransferase (ALT), aspartate aminotransferase (AST), urea, serum creatinine (Scr), and serum uric acid (SUA). Inflammatory Index: Neutrophil count and lymphocyte count were recorded, and the NLR was calculated as a potential marker of systemic inflammation. Lifestyle and Comorbidities: Smoking and drinking status were categorized as “Yes” or “No.” Previous medical history, including hypertension (HTN), type 2 diabetes mellitus (T2DM), and non-alcoholic fatty liver disease (NAFLD), were documented based on previous clinical diagnoses or current diagnostic criteria. For clarity, variables in the revised tables were reorganized into four categories: demographic and anthropometric variables, lifestyle and comorbidity variables, biochemical markers, and inflammatory index. This grouping was intended to separate routine clinical domains and improve readability.

### 2.4 ML model construction and training

The study cohort (*n* = 852) was randomly stratified into a training set (*n* = 598) and an internal validation set (*n* = 254) at a ratio of 7:3 to ensure a balanced distribution of outcome events. The training set was utilized for feature selection and model development, while the validation set was reserved for independent internal performance evaluation. No external validation cohort was available in the present study; therefore, the validation results should be interpreted as internal validation rather than evidence of transportability to other hospitals, regions, or ethnic populations.

Feature selection was performed using LASSO regression within the training data to identify predictive variables and mitigate overfitting. Subsequently, five machine learning algorithms were constructed: LR, SVM, DT, RF, and XGBoost. During the training phase, a 5-fold cross-validation strategy combined with grid search was implemented for hyperparameter optimization. To reduce the risk of data leakage, model tuning and feature selection were conducted without using the internal validation set; the validation set was used only once for final model evaluation. Nested cross-validation was not performed, which is acknowledged as a methodological limitation because it may provide a less conservative estimate of tuning-related uncertainty.

The LASSO penalty parameter (lambda) was selected by 10-fold cross-validation using the lambda.min criterion, defined as the lambda value that produced the minimum mean cross-validated error. At this lambda value, 12 variables retained non-zero coefficients and were entered into subsequent model construction. We chose lambda.min rather than the more parsimonious lambda.1se rule because the objective was to preserve clinically relevant predictors while still reducing dimensionality from the original 19 variables. The exact R code used for data preprocessing, LASSO feature selection, model training, performance evaluation, calibration, DCA, SHAP analysis, and RCS analysis will be provided as to improve transparency and reproducibility.

### 2.5 Model evaluation, validation and interpretation

The discriminative power of the models was assessed in the validation set using the Area Under the Receiver Operating Characteristic (ROC) Curve (AUC). Additional performance metrics, including accuracy, specificity, sensitivity, and the F1-score, were calculated.

Calibration curves were generated to evaluate the agreement between the predicted probabilities and actual observations. Furthermore, Decision Curve Analysis (DCA) was performed to determine the clinical net benefit and utility of the models across different threshold probabilities. To provide clinical interpretability, the SHAP framework was employed to quantify feature contributions to model output. SHAP values were interpreted as model-based associations rather than causal effects, because the retrospective cross-sectional design cannot establish temporality or biological causation.

### 2.6 Statistical analysis tools

All statistical analyses and model developments were performed using R software (version 4.3.2). For baseline characterization, continuous variables with a normal distribution were expressed as mean ± standard deviation (SD) and compared using the Student’s *t*-test. Non-normally distributed data were expressed as median (interquartile range, IQR) and compared using the Mann–Whitney U test. Categorical variables were expressed as frequencies (percentages) and analyzed using the Chi-square test or Fisher’s exact test. A two-tailed *p*-value < 0.05 was considered statistically significant. To further explore the potential non-linear or dose–response relationship between variables and the risk of OP, restricted cubic spline (RCS) analysis was performed with four knots (at the 5th, 35th, 65th, and 95th percentiles). These models were adjusted for the same set of covariates used in the primary multivariable LR. Subgroup-specific RCS analysis by sex was also conducted to identify potential heterogeneities in risk patterns. The main R packages used included glmnet for LASSO regression, caret for data partitioning and model training, randomForest for RF, e1071 for SVM, rpart for DT, xgboost for XGBoost, pROC for ROC/AUC analysis, rms for calibration and restricted cubic spline analysis, rmda for decision curve analysis, and SHAPforxgboost/fastshap for model interpretation. The complete annotated R script is provided.

## 3 Results

### 3.1 Baseline characteristics and group comparisons

A total of 852 older people (age ≥60) were included in this study. The cohort was randomly partitioned into a training set (*n* = 598) and a validation set (*n* = 254) at a 7:3 ratio. As summarized in Table 1, the baseline demographic, lifestyle, and biochemical profiles were well-balanced between the training and validation set. Except for NLR (*p* = 0.029) and FPG (*p* = 0.008), no statistically significant differences were observed across most variables, including age, BMI, sex distribution, and comorbidities (all *p* > 0.05). This consistency provides a solid foundation for the subsequent development of a stable and generalizable LR model, effectively minimizing the risk of model bias between the training and verification phases.

**Table 1:** Baseline characteristics of the study population in the training and validation sets.

Variables	Total (*n* = 852)	Training set (*n* = 598)	Validation set (*n* = 254)	t/Z/χ^2^	*P*
Demographic and anthropometric variables					
Age	65.00 (62.00, 68.00)	65.00 (62.00, 68.00)	65.00 (62.00, 68.00)	−0.958	0.338
Sex				0.430	0.512
Female	492 (57.75)	341 (57.02)	151 (59.45)		
Male	360 (42.25)	257 (42.98)	103 (40.55)		
BMI	25.72 (23.30, 27.79)	25.73 (23.28, 27.89)	25.66 (23.59, 27.41)	−0.352	0.725
Lifestyle and comorbidity variables					
Smoking				0.028	0.868
No	732 (85.92)	513 (85.79)	219 (86.22)		
Yes	120 (14.08)	85 (14.21)	35 (13.78)		
Drinking				0.391	0.532
No	666 (78.17)	464 (77.59)	202 (79.53)		
Yes	186 (21.83)	134 (22.41)	52 (20.47)		
HTN				0.498	0.480
No	545 (63.97)	378 (63.21)	167 (65.75)		
Yes	307 (36.03)	220 (36.79)	87 (34.25)		
T2DM				3.096	0.078
No	741 (86.97)	528 (88.29)	213 (83.86)		
Yes	111 (13.03)	70 (11.71)	41 (16.14)		
NAFLD				0.002	0.961
No	539 (63.26)	378 (63.21)	161 (63.39)		
Yes	313 (36.74)	220 (36.79)	93 (36.61)		
OP				0.003	0.955
No	606 (71.13)	425 (71.07)	181 (71.26)		
Yes	246 (28.87)	173 (28.93)	73 (28.74)		
Biochemical markers					
LDL	3.05 ± 0.88	3.03 ± 0.88	3.08 ± 0.88	−0.703	0.482
TC	5.00 ± 1.02	4.99 ± 1.02	5.04 ± 1.03	−0.639	0.523
TG	1.29 (0.97, 1.79)	1.24 (0.97, 1.78)	1.35 (0.99, 1.79)	−1.390	0.164
HDL	1.42 (1.25, 1.67)	1.42 (1.25, 1.65)	1.38 (1.22, 1.70)	−0.481	0.630
FPG	5.39 (4.97, 6.09)	5.34 (4.91, 5.99)	5.62 (5.03, 6.42)	−2.660	0.008
Hb	137.00 (130.00, 147.00)	137.00 (130.00, 147.00)	138.00 (129.00, 147.00)	−0.332	0.740
Urea	4.90 (4.15, 5.73)	4.99 (4.16, 5.74)	4.72 (4.08, 5.69)	−1.335	0.182
Scr	72.80 (62.68, 83.10)	72.85 (62.82, 82.77)	72.60 (62.50, 83.55)	−0.035	0.972
SUA	315.50 (267.00, 375.25)	316.00 (265.00, 376.00)	314.00 (271.25, 373.75)	−0.282	0.778
ALT	18.10 (14.47, 23.83)	18.20 (14.62, 24.30)	17.80 (13.53, 23.17)	−1.466	0.143
AST	22.80 (19.67, 26.30)	23.00 (19.83, 26.30)	22.05 (19.12, 26.17)	−1.692	0.091
Inflammatory index					
NLR	1.62 (1.29, 2.20)	1.64 (1.31, 2.22)	1.56 (1.18, 2.10)	−2.181	0.029

Data are presented as mean ± standard deviation (SD) for normally distributed continuous variables (evaluated by *t*-test), median (interquartile range, Q1, Q3) for non-normally distributed continuous variables (evaluated by Mann–Whitney U test), and frequency (percentage) for categorical variables (evaluated by Chi-square test).

### 3.2 Clinical comparisons between OP and non-OP groups

In the total cohort, participants were categorized into the OP group (*n* = 246) and the Non-OP group (*n* = 606), representing a prevalence of 28.87%. Univariate analysis revealed significant disparities between the two groups. Specifically, participants in the OP group were older (67.50 vs. 64.00 years, *p* < 0.001), had a lower BMI (24.91 vs. 26.11 kg/m^2^, *p* < 0.001), and exhibited lower levels of Hb, Scr, and SUA (all *p* < 0.001). Notably, the OP group showed significantly higher HDL levels (1.54 vs. 1.37 mmol/L, *p* < 0.001) and a higher proportion of females (79.27% vs. 49.01%, *p* < 0.001). Lifestyle factors such as smoking and drinking were also significantly associated with OP status (both *p* < 0.001) (Table 2). These distinct clinical and biochemical profiles provided the requisite evidence-based rationale for the feature selection process, ensuring the clinical interpretability of the final LR-based predictive framework.

**Table 2:** Comparison of clinical characteristics and biochemical indicators between OP and non-OP groups.

Variables	Total (*n* = 852)	Non-OP (*n* = 606)	OP (*n* = 246)	t/Z/χ^2^	*P*
Demographic and anthropometric variables					
Age	65.00 (62.00, 68.00)	64.00 (61.00, 67.00)	67.50 (64.00, 72.00)	−7.885	<0.001
Sex				65.656	<0.001
Female	492 (57.75)	297 (49.01)	195 (79.27)		
Male	360 (42.25)	309 (50.99)	51 (20.73)		
BMI	25.72 (23.30, 27.79)	26.11 (23.90, 28.19)	24.91 (22.33, 26.96)	−5.782	<0.001
Lifestyle and comorbidity variables					
Smoking				22.133	<0.001
No	732 (85.92)	499 (82.34)	233 (94.72)		
Yes	120 (14.08)	107 (17.66)	13 (5.28)		
Drinking				38.045	<0.001
No	666 (78.17)	440 (72.61)	226 (91.87)		
Yes	186 (21.83)	166 (27.39)	20 (8.13)		
HTN				0.534	0.465
No	545 (63.97)	383 (63.20)	162 (65.85)		
Yes	307 (36.03)	223 (36.80)	84 (34.15)		
T2DM				4.130	0.042
No	741 (86.97)	518 (85.48)	223 (90.65)		
Yes	111 (13.03)	88 (14.52)	23 (9.35)		
NAFLD				4.398	0.036
No	539 (63.26)	370 (61.06)	169 (68.70)		
Yes	313 (36.74)	236 (38.94)	77 (31.30)		
OP				852.000	<0.001
No	606 (71.13)	606 (100.00)	0 (0.00)		
Yes	246 (28.87)	0 (0.00)	246 (100.00)		
Biochemical markers					
LDL	3.05 ± 0.88	3.01 ± 0.88	3.12 ± 0.89	−1.627	0.104
TC	5.00 ± 1.02	4.95 ± 1.02	5.14 ± 1.01	−2.468	0.014
TG	1.29 (0.97, 1.79)	1.31 (1.00, 1.79)	1.22 (0.93, 1.77)	−0.911	0.362
HDL	1.42 (1.25, 1.67)	1.37 (1.19, 1.62)	1.54 (1.34, 1.77)	−5.698	<0.001
FPG	5.39 (4.97, 6.09)	5.45 (5.03, 6.28)	5.24 (4.81, 5.88)	−3.248	<0.001
Hb	137.00 (130.00, 147.00)	140.00 (132.00, 148.75)	133.00 (126.25, 141.00)	−5.867	<0.001
Urea	4.90 (4.15, 5.73)	4.90 (4.15, 5.74)	4.90 (4.13, 5.70)	−0.510	0.610
Scr	72.80 (62.68, 83.10)	74.70 (64.73, 84.18)	67.55 (58.52, 77.47)	−6.230	<0.001
SUA	315.50 (267.00, 375.25)	320.50 (274.00, 381.00)	297.00 (247.25, 340.75)	−4.605	<0.001
ALT	18.10 (14.47, 23.83)	18.55 (14.70, 24.45)	17.70 (13.90, 22.70)	−2.070	0.038
AST	22.80 (19.67, 26.30)	22.40 (19.42, 26.20)	23.00 (20.50, 26.98)	−1.358	0.175
Inflammatory index					
NLR	1.62 (1.29, 2.20)	1.63 (1.31, 2.21)	1.60 (1.18, 2.17)	−1.299	0.194

Data are presented as mean ± standard deviation (SD) for normally distributed continuous variables (evaluated by *t*-test), median (interquartile range, Q1, Q3) for non-normally distributed continuous variables (evaluated by Mann–Whitney U test), and frequency (percentage) for categorical variables (evaluated by Chi-square test).

### 3.3 Feature selection and model performance evaluation

**Figure 2:**
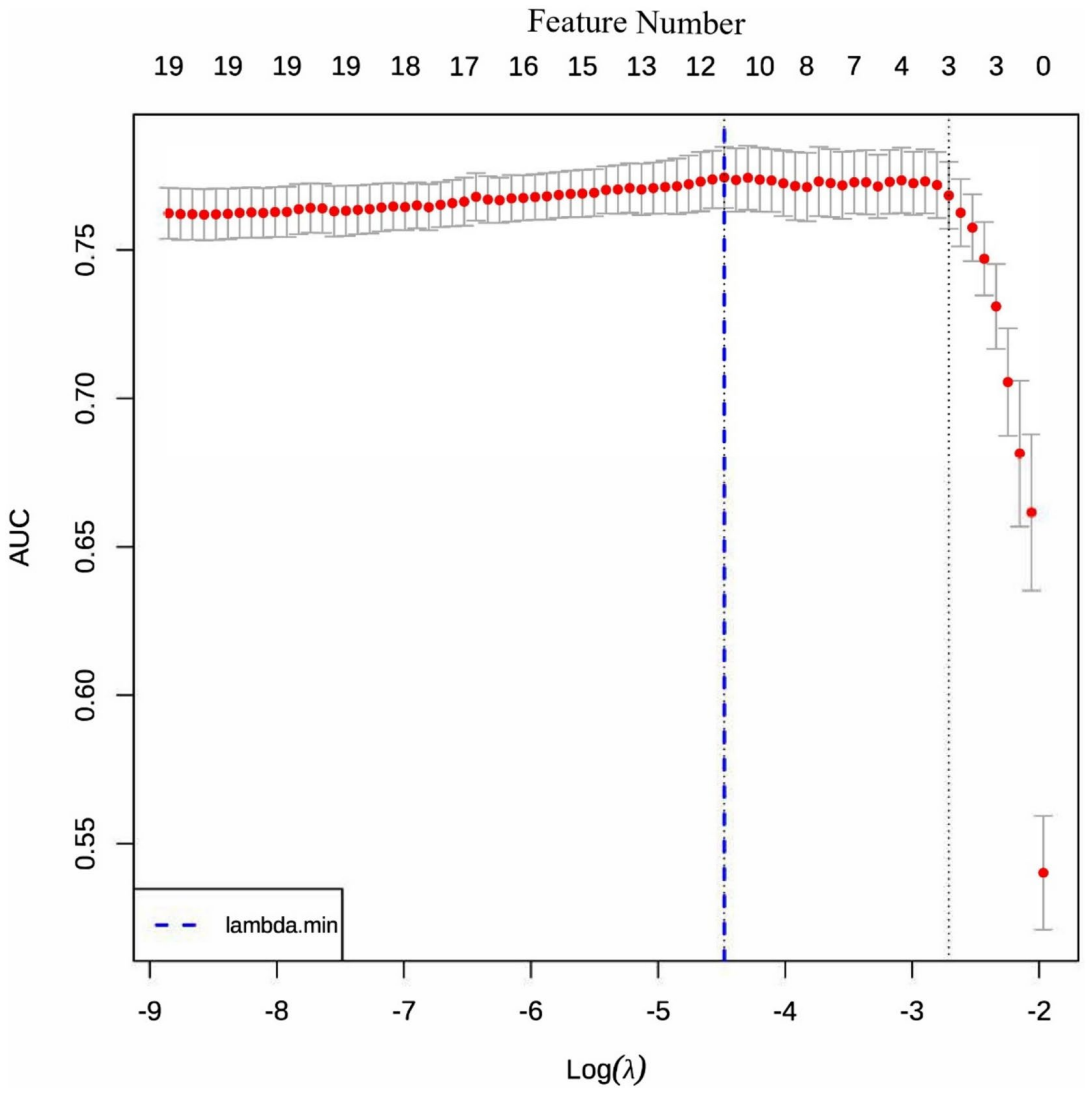
Feature selection using the LASSO binary logistic regression model.

**Figure 3:**
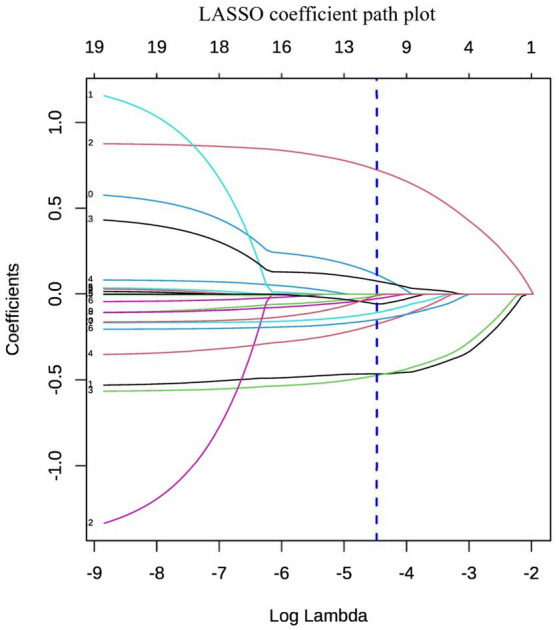
LASSO coefficient profiles of the 19 features. Selection of the optimal *lambda* (*λ*) in the LASSO model via 10-fold cross-validation. The vertical blue dashed line indicates the *λ* (min) value that yields the minimum mean cross-validated error. The 12 retained features correspond to the non-zero coefficients at lambda.min.

**Figure 4:**
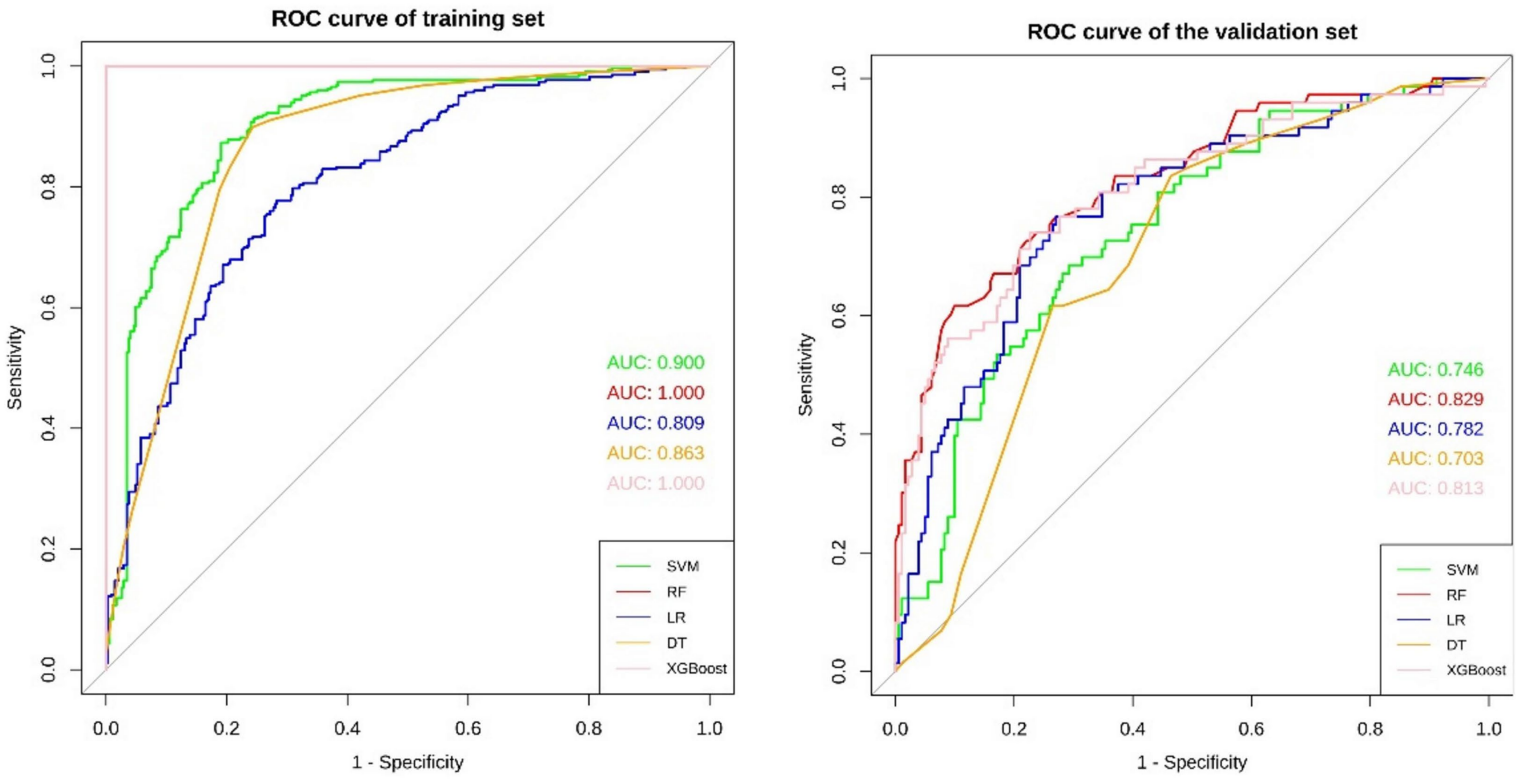
ROC curves for the five machine learning models in the training set (left) and validation set (right).

Using 10-fold cross-validation, the LASSO model selected lambda.min as the penalty parameter because it minimized the mean cross-validated prediction error. At lambda.min, 12 of the 19 candidate variables had non-zero coefficients and were retained for downstream modelling (Figures 2, 3). This selection reflected a balance between prediction performance and dimensionality reduction. Among the five ML models evaluated, RF and XGBoost achieved perfect discrimination in the training set (AUC = 1.000), which strongly suggests overfitting despite their acceptable validation-set AUCs. In contrast, the LR model exhibited more balanced performance, with an AUC of 0.809 in the training set and 0.782 in the internal validation set. Although RF and XGBoost showed numerically higher validation AUCs, the LR model was selected as the preferred model because of its lower apparent overfitting, simpler structure, and greater clinical interpretability (Table 3; Figure 4).

**Table 3:** Performance metrics of five machine learning models in the training and validation sets.

Model	Data set	AUC	Sensitivity	Specificity	Accuracy	F1 Score
LR	Training set	0.809	0.763	0.723	0.743	0.748
	Validation set	0.782	0.767	0.702	0.720	0.612
RF	Training set	1.000	1.000	1.000	1.000	1.000
	Validation set	0.829	0.740	0.762	0.756	0.635
DT	Training set	0.863	0.899	0.757	0.828	0.839
	Validation set	0.703	0.644	0.641	0.642	0.508
SVM	Training set	0.900	0.873	0.806	0.840	0.845
	Validation set	0.746	0.699	0.669	0.677	0.554
XGBoost	Training set	1.000	1.000	1.000	1.000	1.000
	Validation set	0.813	0.726	0.773	0.760	0.635

### 3.4 Calibration and clinical utility

**Figure 5:**
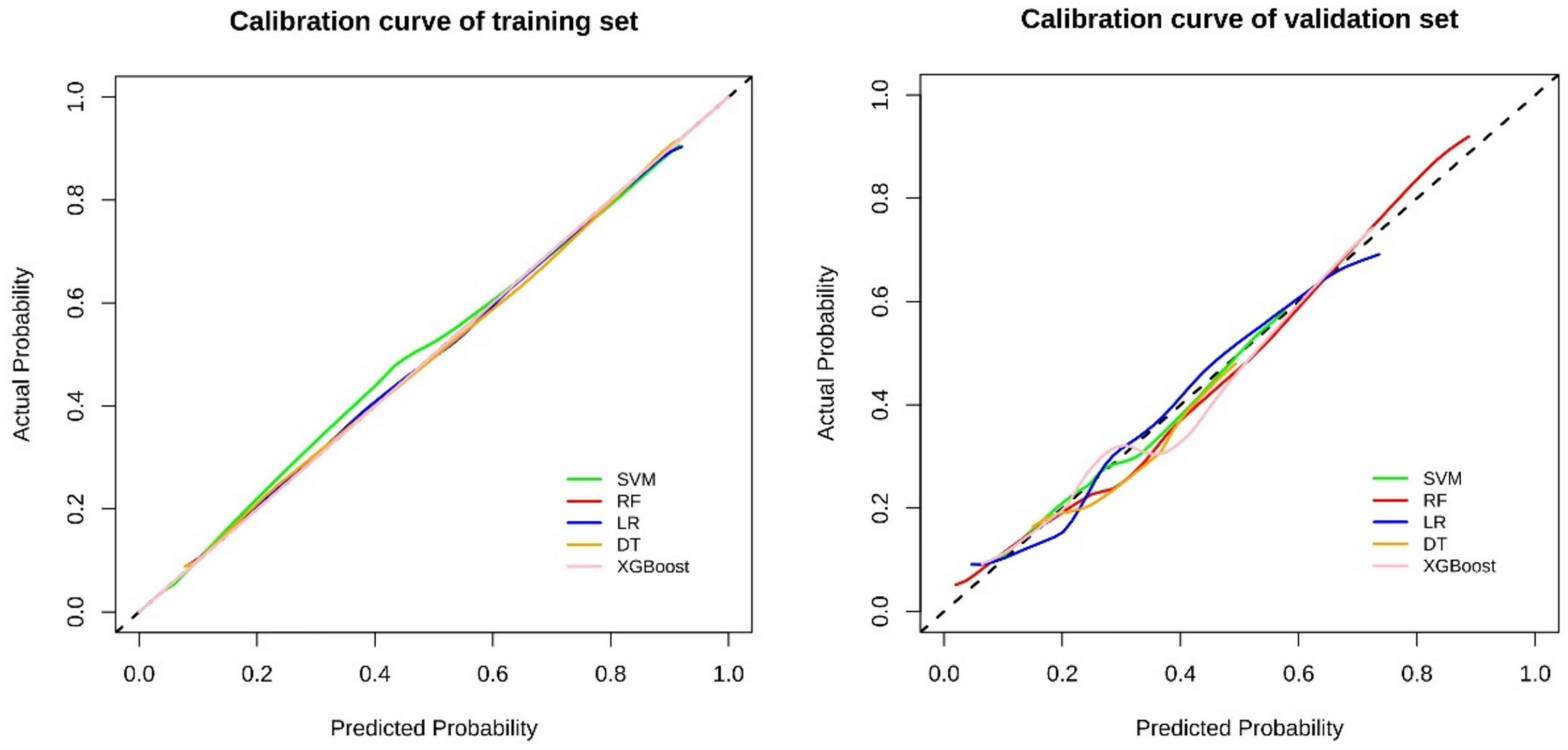
Calibration curves of the five models. The *x*-axis represents the predicted probability of OP, and the *y*-axis represents the actual observed probability. The diagonal dashed line represents a perfect prediction.

**Figure 6:**
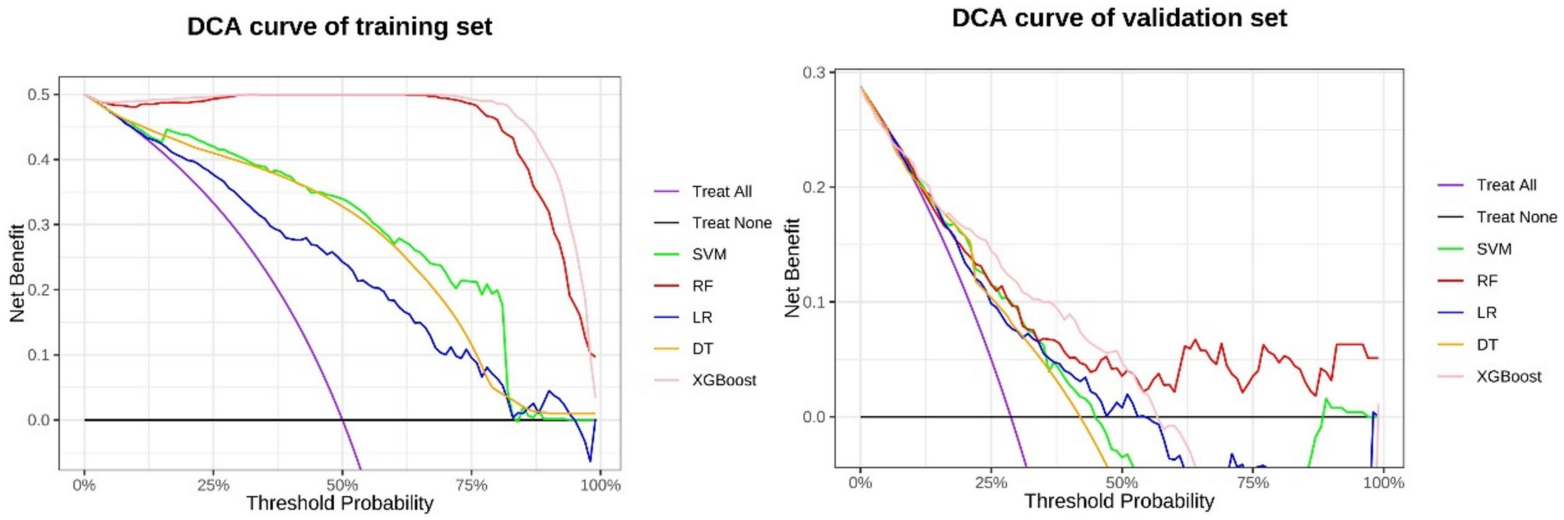
DCA for the five models. The *x*-axis represents the threshold probability of OP, and the *y*-axis measures the net benefit.

Calibration curves for the LR model showed high agreement between predicted and observed OP risks in both sets (Figure 5). Most importantly, DCA demonstrated that the LR model provided a substantial and consistent clinical net benefit over a wide range of threshold probabilities. Given its stability, lack of overfitting, and superior clinical interpretability, the LR model was identified as the optimal tool for predicting OP in this population of older people (Figure 6).

### 3.5 SHAP interpretation and non-linear association analysis

**Figure 7:**
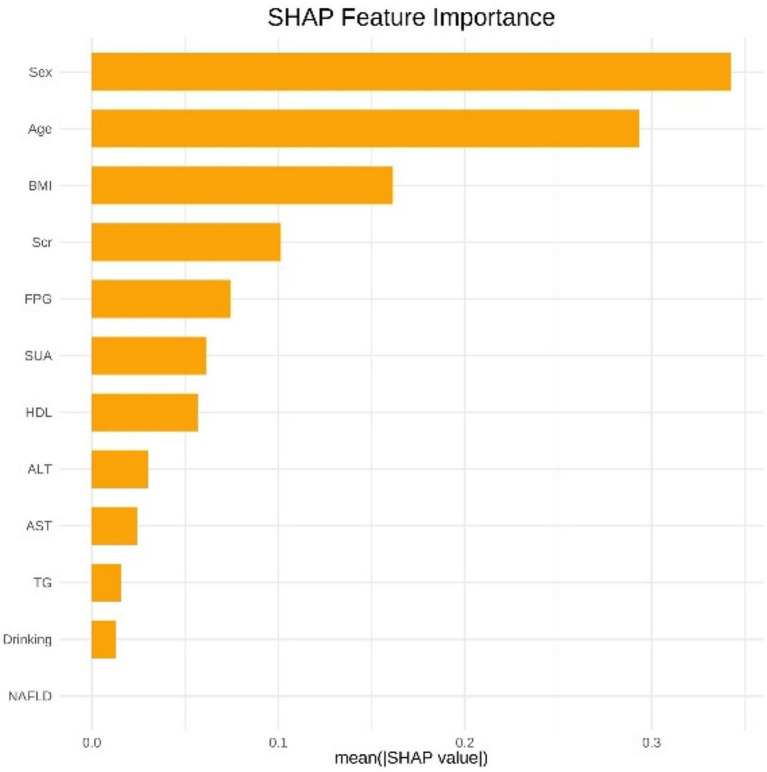
Global feature importance based on SHAP values. The bar chart illustrates the average absolute SHAP values for each clinical variable, representing their global contribution to the machine learning model. Sex, Age, and BMI were identified as the top three predictors, followed by biochemical markers including FPG, Scr, and SUA. These variables contributed meaningfully to model-based risk stratification, but SHAP rankings do not imply causal effects.

**Figure 8:**
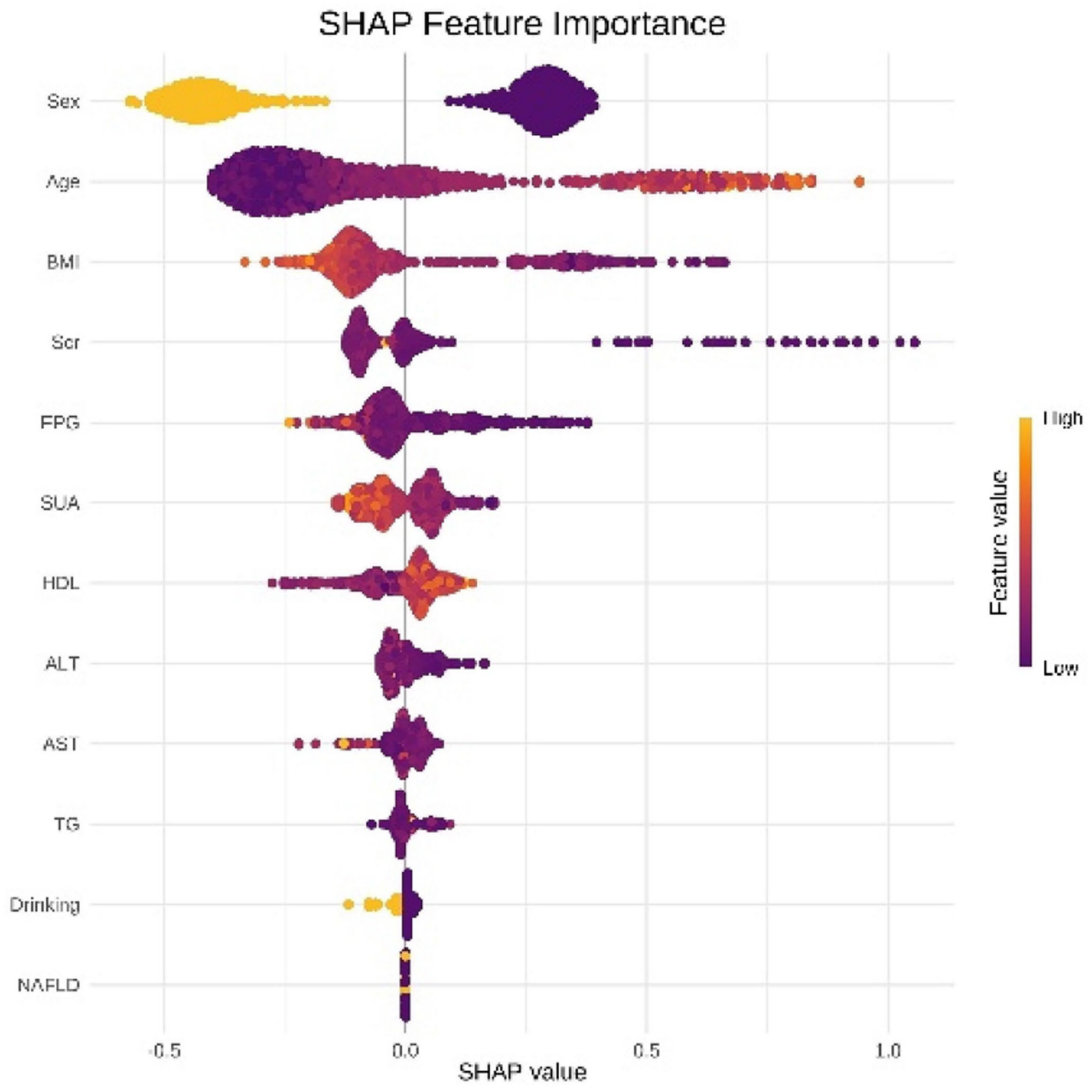
SHAP summary plot of feature impacts on osteoporosis risk. The beeswarm plot displays the distribution of SHAP values for each feature across the cohort. Each dot represents an individual participant, with colors indicating feature levels (red/yellow for high, purple/blue for low). Positive SHAP values denote increased model-predicted risk of osteoporosis. Results suggest that female sex, advanced age, and lower BMI were associated with higher predicted risk. Lower levels of Scr and SUA, along with lower FPG, also contributed to increased predicted risk in the model.

To elucidate the internal logic of the predictive framework, SHAP analysis was conducted on the total cohort as an exploratory interpretation of model behavior (Figures 7, 8). Sex was the most significant feature, with the female group exhibiting a higher predicted risk profile. Age showed a positive association with predicted risk, while BMI exhibited an inverse association (higher BMI associated with lower SHAP values). Among the biochemical markers, Scr and SUA displayed a “lower-value, higher-risk” pattern. Specifically, participants with lower Scr and SUA concentrations tended to receive higher model-predicted OP risk. Similarly, lower FPG levels within this cohort of older people were associated with increased SHAP values. These findings should be interpreted as predictive associations rather than evidence that these markers directly cause osteoporosis.

**Figure 9:**
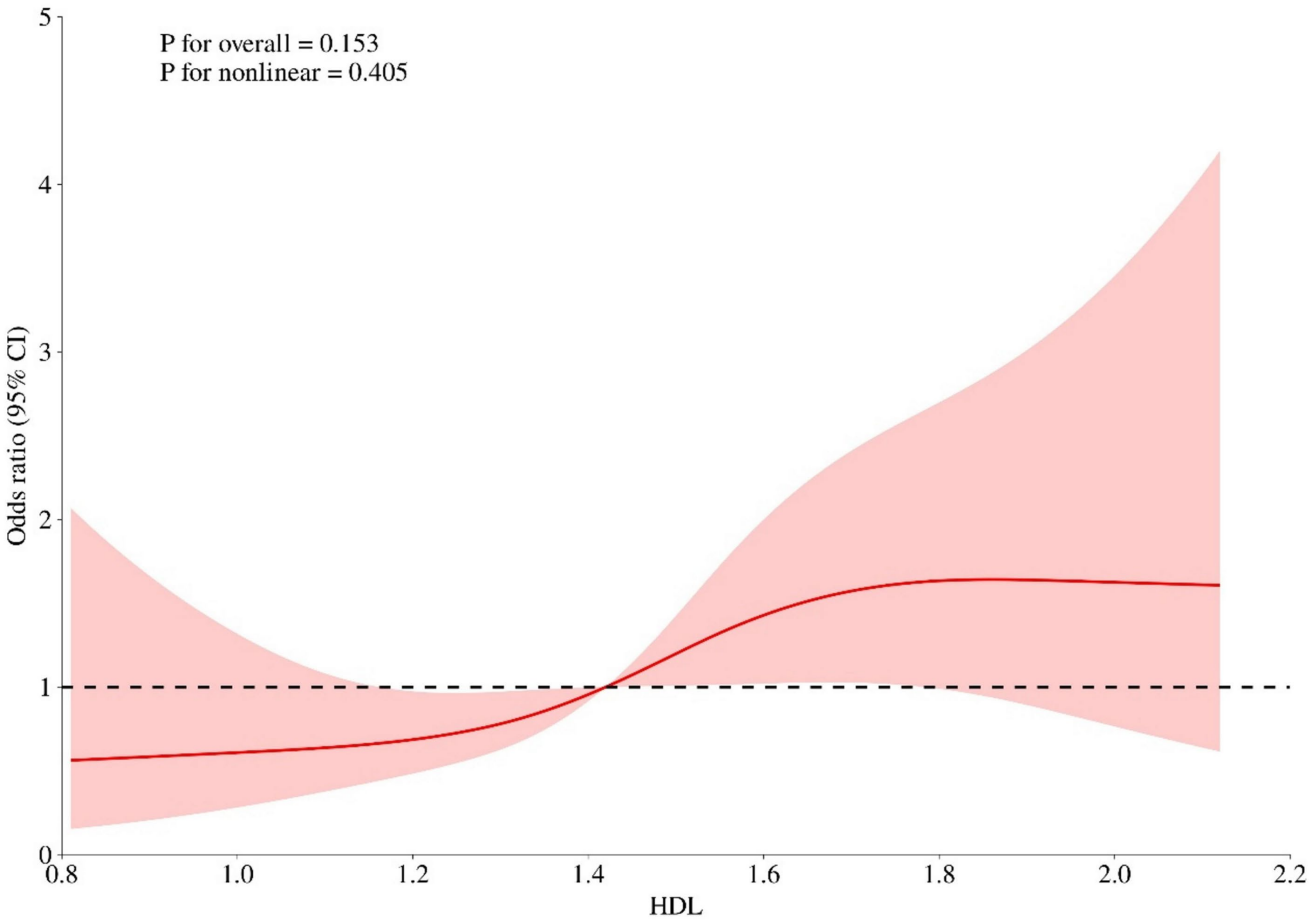
RCS plots illustrating the association between HDL-C and the risk of OP. Data are presented as Odds Ratios (solid lines) with 95%CI (shade areas) adjusted for age, BMI, FPG, Scr, SUA, TC, TG and LDL-C.

To further explore the dose–response relationship between biochemical markers and bone health, multivariable-adjusted RCS analysis was performed (Figure 9). The results suggested a linear upward trend between HDL-C levels and OP risk (P for non-linearity >0.05), consistent with the model-based importance of lipid profiles. However, the overall association was not statistically significant in the total population (P for overall = 0.153). Subgroup analysis by sex showed a stronger trend in females (OR = 3.35, 95% CI: 0.86–13.04; P for overall = 0.081), whereas no clear association was observed in males (OR = 1.67, 95% CI: 0.18–15.19; P for overall = 0.650). Because the female subgroup result did not reach conventional statistical significance, it should be regarded as hypothesis-generating rather than confirmatory.

## 4 Discussion

In this study, we developed and internally validated an ML framework to predict the risk of OP in older people. By integrating multi-dimensional clinical data, the LR model demonstrated comparatively stable discrimination and calibration in the internal validation set. Importantly, the model should be viewed as a pre-screening aid that may help prioritize older people for DXA assessment and preventive counseling, rather than as a replacement for DXA-based diagnosis. SHAP analysis provided a transparent map of feature contributions and highlighted associations involving FPG, Scr, SUA, and HDL-C beyond traditional predictors such as sex, age, and BMI; however, these associations should be interpreted cautiously because causal inference is not supported by the study design.

Our results identify age, sex, and BMI as the most potent biological predictors of bone loss, a finding highly consistent with global epidemiological trends and further quantified through SHAP analysis (16–18). Advanced age was recognized as the primary driver of bone deterioration (16). Modern perspectives no longer view this transition as a simple “wear-and-tear” process but rather because of cellular senescence within the bone microenvironment. Recent research by Wang D et al. (19) demonstrated that aging triggers the senescence-associated secretory phenotype (SASP) in osteocytes, creating a pro-inflammatory milieu that over-activates osteoclasts. Our findings also corroborate the work of Di Cicco G et al. (20), who reported that the age-related imbalance in the RANK/RANKL/OPG signaling pathway in cohorts over 60 leads to a definitive shift toward bone resorption over formation, a trend clearly reflected in the high SHAP values attributed to age in our model.

Regarding gender disparities, the significantly higher prevalence of OP in females in our cohort aligns with the physiological decline in estrogen post-menopause (21). Estrogen deficiency removes the “brake” on osteoclastic activity; however, recent research by Luo J et al. (22) further emphasizes that older women exhibit higher sensitivity to oxidative stress-induced bone loss compared to men. While previous studies focused largely on the perimenopausal period, our results indicate that for individuals aged 60 and older, this “estrogen gap” and its associated cascades remain the dominant risk factors well into senescence.

Furthermore, SHAP analysis confirmed that BMI exerts a significant bone-protective effect in older people, strongly supporting the “obesity paradox” in skeletal health. While high BMI is a risk factor for cardiovascular disease, its role in the skeletal system is multi-dimensional (23, 24). Mechanistically, mechanical mechanotransduction induced by gravitational loading on the skeletal framework stimulates osteoblast activity through mechanotransduction pathways. Simultaneously, adipose tissue acts as an extra-gonadal reservoir, where aromatase converts androgens into estrogens, serving as a primary source of bone-protective hormones in geriatric populations (25). Our results align with Liu Y et al. (26), suggesting that maintaining a BMI in the “overweight” range (24.0–27.9 kg/m^2^) is associated with the lowest OP risk in older people. However, it is vital to distinguish between overall BMI and muscle mass. Given the high SHAP ranking of Scr (a proxy for muscle mass), the protective effect of BMI likely stems from the synergy between mechanical loading and a robust muscle scaffold rather than mere adiposity (26).

This study observed an association between lower FPG and higher model-predicted OP risk in this cohort of older people, which should be interpreted in the context of the study population rather than as evidence that low glucose directly causes OP (27). In non-diabetic older people, FPG may partly reflect nutritional reserve, frailty, or anabolic status.

In practical terms, this finding should be understood as a frailty-related signal rather than a recommendation to maintain low glucose levels. Among older adults without overt diabetes, relatively low fasting glucose may coexist with reduced appetite, lower caloric/protein intake, sarcopenia, and lower anabolic hormone activity (28–30). These conditions may weaken osteoblast function, reduce muscle-bone mechanical loading, and increase vulnerability to bone loss (31–33). This interpretation is supported by evidence that glucose uptake is required for osteoblast differentiation and bone formation (31), inadequate protein intake is associated with sarcopenia in older adults (28), malnutrition contributes to falls and osteoporotic fractures (32), and lower IGF-1 levels are associated with reduced BMD or femoral bone loss in older populations (29, 30). Because medication use, detailed nutritional measures, and longitudinal glycemic trajectories were unavailable, the observed FPG pattern should be treated as a hypothesis-generating predictive association.

Osteoblast differentiation and matrix synthesis are energetically demanding processes. Lower FPG may reflect a state of chronic negative energy balance or “malnutrition-related frailty.” Consistent with established bioenergetic models (34, 35), osteoblasts exhibit an exceptional reliance on glucose as their primary biosynthetic substrate. Under conditions of low-glucose stress, metabolic sensors such as AMP-activated protein kinase are activated, subsequently antagonizing the mTORC1 signaling pathway to downregulate protein translation and collagen matrix synthesis, ultimately impairing bone formation. Chronic low-level glycemia is often accompanied by reduced secretion of insulin-like growth factor-1 (IGF-1) (36). Mazziotti G et al. (37) noted that IGF-1 is important regulators of bone remodelling and metabolism and has an essential role in the achievement and maintenance of bone mass throughout life. Low FPG often signals the decline of the “growth hormone-IGF-1 axis,” leading to insufficient bone formation (38). This anabolic impairment due to a lack of “metabolic fuel” is a significant, yet often hidden, cause of bone loss in older people.

SHAP results indicated that low Scr contributed to higher model-predicted risk, plausibly reflecting reduced muscle mass rather than a direct causal effect of creatinine itself. Muscle and bone engage in cross-organ communication via biochemical and mechanical signals (39). Recent studies have demonstrated that muscle-derived factors such as irisin may exert protective effects on bone cells (40). Low Scr may therefore act as a surrogate marker of sarcopenia or reduced muscle reserve, which can weaken mechanical support and bone-forming signals (41–43). This finding supports the clinical relevance of considering the bone-muscle unit as an integrated system in OP risk assessment in older people.

The “low-SUA, high-risk” pattern revealed by SHAP provides deep insights into the disruption of redox homeostasis in geriatric OP, a finding consistent with previous literature (44–46). The mechanism underlying this association is primarily driven by oxidative stress, a central factor in the pathogenesis of OP. Oxidative stress is a central mechanism in the pathogenesis of OP. As the predominant antioxidant in human plasma, SUA neutralizes excessive reactive oxygen species (ROS). Under typical conditions, excessive ROS inhibits the proliferation and differentiation of osteoblasts by suppressing key transcription factors, specifically Runx2 and Osterix (46). Simultaneously, it facilitates osteoclastogenesis by upregulating markers such as c-Fos and NFATc1, ultimately exacerbating bone destruction (46). SUA counteracts this oxidative process, thereby mitigating bone damage and preserving osteogenic function. Furthermore, elevated SUA levels are directly associated with a decline in bone resorption markers, effectively lowering the overall bone turnover rate (46).

The association between lipid profiles and bone homeostasis observed in this study may be explained by several interconnected pathways (47, 48). Elevated HDL-C may serve as a surrogate marker for changes in sex-hormone environment or broader lipid metabolism, both of which may relate to bone mineral density (47). Metabolic dysregulation characterized by insulin resistance and altered adipokine signaling (e.g., leptin and adiponectin) may further disrupt the lipid-bone axis (49, 50). Additionally, lipid oxidation products may influence vascular calcification and bone formation pathways (51). However, because the HDL-C association in the female subgroup was only marginal and statistically non-significant, this finding should be described as exploratory and not used alone for clinical decision-making.

From a clinical workflow perspective, the model may be most useful in routine physical examination or primary care settings as a first-step risk stratification tool. Older people, particularly women, with a combination of advanced age, lower BMI, low-normal Scr or SUA, and elevated model-predicted risk could be prioritized for DXA testing, nutritional assessment, exercise guidance, and fall-risk counseling. This proposed use is complementary to DXA and should be confirmed through external validation and implementation studies before routine adoption.

Despite the performance of our predictive framework, several limitations should be acknowledged. First, this study used a retrospective cross-sectional design, which precludes definitive causal conclusions between the identified biochemical markers and osteoporosis; long-term prospective studies are required to validate temporal relationships. Second, no formal *a priori* sample size calculation was conducted, and the sample size was smaller than some multicenter Chinese prediction studies, although all eligible participants with complete DXA and clinical data during the study period were included and the event-per-variable ratio was acceptable after feature selection. Third, as a single-center study conducted at an urban tertiary-hospital physical examination center in Shijiazhuang, Hebei Province, the cohort may be subject to selection bias. Individuals attending health examinations are often volunteers or participants in employer-organized screening programs, and they may be more health-conscious, more likely to have stable access to medical services, and different from symptomatic hospital patients or community residents who do not undergo routine screening. Fourth, the population was predominantly Han Chinese and drawn from an urban/suburban catchment area, which limits generalizability to rural populations, minority ethnic groups, other regions of China, and international populations. Fifth, the study used an internal 7:3 split rather than true external validation, so the observed validation performance should be interpreted as internal validation only. Sixth, although hyperparameter tuning was restricted to the training data using cross-validation and grid search, nested cross-validation was not performed; therefore, residual optimism in model comparison cannot be fully excluded. Seventh, while sex-stratified RCS analysis suggested an elevated HDL-C-related trend in females (OR = 3.35, 95% CI: 0.86–13.04; *p* = 0.081), this association was not statistically significant and should be considered hypothesis-generating. Future multi-center studies with external validation cohorts and prospective follow-up are needed to confirm model transportability, refine the clinical threshold for DXA referral, and clarify the biological meaning of these metabolic associations.

## 5 Conclusion

This study developed an interpretable LR-based model for OP risk prediction in older people using routine clinical indicators. The model highlighted age, sex, BMI, and several biochemical markers as useful predictors and may support pre-screening decisions in primary care or physical examination settings where DXA resources are limited. However, the findings represent internally validated predictive associations rather than causal relationships. External multi-center validation and prospective implementation studies are needed before the model can be recommended for routine clinical deployment.

## Data Availability

The original contributions presented in the study are included in the article; further inquiries can be directed to the corresponding author.
